# Multi-Functional Laccase Immobilized Hydrogel Microparticles for Efficient Removal of Bisphenol A

**DOI:** 10.3390/ma12050704

**Published:** 2019-02-27

**Authors:** Mingyue Piao, Donglei Zou, Yuesuo Yang, Xianghao Ren, Chuanyu Qin, Yunxian Piao

**Affiliations:** 1 Key Laboratory of Groundwater Resources and Environment (Jilin University), Ministry of Education, Jilin Provincial Key Laboratory of Water Resources and Environment, College of New Energy and Environment, Jilin University, Changchun 130021, China; piaomingyue1985@163.com (M.P.); zoudl@jlu.edu.cn (D.Z.); yangyuesuo@jlu.edu.cn (Y.Y.); qincyu@jlu.edu.cn (C.Q.); 2 College of Environmental Science and Engineering, Jilin Normoal University, 1301 Haifeng Road, Siping 136000, China; 3 Key Laboratory of Eco-restoration of Regional Contaminated Environment, Ministry of Education, Shenyang University, Shenyang 110044, China; 4 Key Laboratory of Urban Storm water System and Water Environment, Ministry of Education, School of Environment and Energy Engineering, Beijing University of Civil Engineering and Architecture, Beijing 100044, China; Renxianghao@bucea.edu.cn

**Keywords:** hydrogel microparticle, laccase, entrapment, bisphenol a removal

## Abstract

Hghly stable, reusable, and multi-functional biocatalytic microparticles with Laccase (Lac) enzyme (Lac/particles) were synthesized for bisphenol A (BPA) removal from aqueous solution. The Lac/particles were prepared by encapsulating Lac enzymes into poly ethylene glycol (PEG) hydrogel via the UV assisted emulsion polymerization method followed by cross linking with glutaraldehyde (GA). The obtained Lac/particles were spherical and micron sized (137–535 μm), presenting high enzyme entrapment efficiency of 100%, high activity recovery of 18.9%, and great stability at various pHs (3–7) than the free Lac. The Lac/particles could adsorb the BPA into the catalytic particles in a short time, promoting contact between BPA and enzyme, and further enzymatically degrade them without the shaking process and independent surrounding buffer solution. The Lac/particles could be reused for another round BPA adsorption and biotranformation by maintaining over 90% of BPA removal efficiency after seven times reuse. The synergistic effects of adsorption and biocatalytical reaction of Lac/particles have significant values in high efficient and cost-effective BPA removal.

## 1. Introduction

Bisphenol A (BPA) has attracted public concern, because its hormone-like effect on the endocrine system disrupts physiological functions. As an important industrial material, small amounts of BPA inevitably enter the environment during production and processing [1]. Low doses of BPA, even at sub-nanogram levels, can affect human health, causing cancer, diabetes, cardiovascular disease, and many other health problems [2]. Therefore, BPA removal is crucial.

Various methods have been utilized to eliminate BPA in polluted water, including photocatalysis [3], adsorption [4], electrocatalysis [5,6], biodegradation [7], and enzymatic oxidation [8,9]. Enzymatic biocatalysts are especially attractive, because of its mild reaction condition and generating compounds with less estrogenic activity than the parent ones [10,11]. To improve properties of enzymes such as reusability, stability, and robustness, the process of enzyme immobilization is essential [12]. Among various immobilization methods, entrapment is beneficial to preserve the activity and functional structure of the enzyme, because enzymes are physically confined into a host matrix, such as polymeric networks or microcapsules, without extra chemical modification. Additionally, it is advantageous for its mild immobilization condition, highly cross-linked networks, limited denaturation, high immobilization efficiency [13], and enabling transport of low molecular weight compounds through the permeable matrix [14].

Hydrogels are well known for providing biocompatible environments for immobilizing biosubstances in a desired composite structure. It was utilized in various medical and industrial applications, such as drug delivery [15,16], tissue engineering [17], biosensor [18], the food industry [19,20], and production of slow release fertilizers [21], as well as in the adsorptive removal of environment pollutants, such as metal [22,23,24] and dyes [25,26,27]. In addition, hydrogels could also be used as a matrix for enzyme immobilization to improve the performance of the biocatalytic reaction. For example, Koklukaya et al. immobilized Laccase (Lac) in poly (acrylamide-crotonic acid)/sodium alginate, poly (acrylamidecrotonic acid)/K-carrageenan, or poly(acrylamide-citraconic acid)/K-carrageenan hydrogels by the entrapping method for the biotransformation of acid orange [28]. Asgher et al. encapsulated Lac into agar-agar, polyacrylamide, or gelatin hydrogels, then assessed their stability and kinetic properties [29]. These hydrogels endowed Lac with more stability than free Lac, but they should be cut into pieces before use as their pristine form was not suitable for direct application. It was also reported that the spherical chitosan-based or alginate-based hydrogel particles containing Lac could be produced by injecting Lac-gel mixture to a hardening solution, but the production yield was generally low and the particle size was often in millimeters, which may be adverse for mass transfer during pollutant treatment [30,31,32]. As far as we know, hydrogels are not only excellent supports to entrap enzymes, but can also be good adsorbents for some pollutants in water. Sun et al. produced the Lac-PAM-Chitosan that could pre-enrich Malachite Green before enzymatic catalysis, but they should also be crushed into small particles before use in order to reduce mass transfer resistance [33]. 

In view of facilitating easy mass transfer and efficient biocatalytic reaction for pollutants removal, the production of hydrogel biocatalysts with micro size and spherical shaped particles, accompanied with an adsorption property, is of great benefit. In this study, we synthesized Lac entrapped polyethylene glycol (PEG) hydrogel microparticles (Lac/particles) consisting of poly (ethylene glycol) diacrylate (PEGDA) and poly (ethylene glycol) methacrylate (PEGMA) by using a UV assisted emulsion polymerization method. PEG-based hydrogels are particularly attractive because of their excellent biocompatibility, tunable network structure, good toughness, and readily modifiable properties [34]. The emulsion/UV polymerization method is superior because it is easy to perform, requires less arduous equipment, and can produce large batches of microparticles quickly with appropriate sizes, as compared to other methods such as polymerization, microfluidic polymerization, lithographic process, and electro spraying fabrication [35]. Additionally, it would not exhibit drastic changes in volume independent of the variation of temperature, pH, and light [36,37,38,39,40]. One of the critical advantages of using Lac entrapped PEG hydrogel microparticles for BPA removal is reducing the effect of mass transfer resistance between enzyme and substrate due to the small size of particles. Another critical advantage desired is that when PEG hydrogel microparticles were utilized for pollutant removal, the pollutant would be adsorbed and enriched in the hydrogel network, prolonging the contact time between the substrate and enzyme, and enable fast and efficient pollutant removal. 

## 2. Materials and Methods

### 2.1. Chemicals and Materials

Poly (ethylene glycol) diacrylate (PEGDA, MW 575), poly (ethylene glycol) methacrylate (PEGMA, MW 360), 2-hydro-xy-2-methylpropiophenone (HMPP), 2,2′-azino-bis (3-ethylbenzothiazoline-6-sulfonic acid) (ABTS), mineral light oil, Span 80, Tween 20, BPA, glutaraldehyde (GA), fluorescein isothiocyanate (FITC) assay kit, and Pluronic F-127 were purchased from Sigma-Aldrich (St. Louis., MO, USA). Laccase (Lac, 1.07 U/mg) from Trametes versicolor was also purchased from Sigma-Aldrich and was used without further purification. Bradford reagent was purchased from Thermo fisher (Waltham, MA, USA). Other agents were at least of analytical grade. Citric phosphate buffer (50 mM) and ultrapure water were used in all experiments. 

### 2.2. Preparation of Lac/Particles

**Figure 1:**
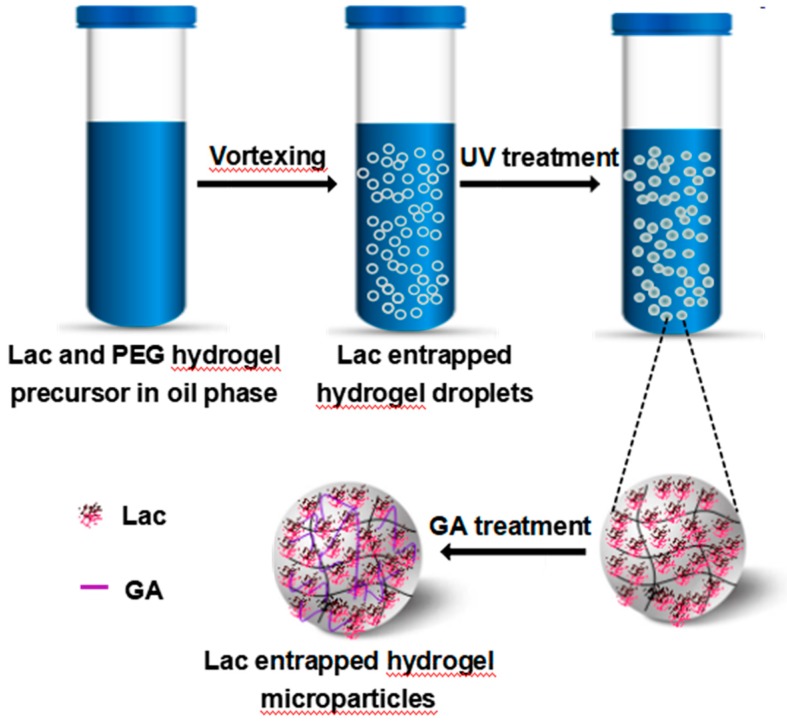
Schematics for the preparation of laccase entrapped polyethylene glycol (PEG) hydrogel microparticles (Lac/particles).

Lac entrapped in hydrogel microparticles (Lac/particles) were prepared using reverse emulsion assisted by UV polymerization, as shown in Figure 1. The crucial step in Lac/particles preparation is the formation of the stable hydrogel microdroplets in the oil phase. Hence, several factors, including the ratio of phase volumes (oil/water), surfactant, and the ratio of PEGDA/PEGMA mixture, were controlled during the experiments. Span 80 (1%) and Tween 20 (1%) were served to prevent reaggregation of the droplets and increase productivity. The ratio of PEGDA/PEGMA was adjusted to 4:1 (v:v), which was not only appropriate to form a spherical shape, but also beneficial to maintain rigidness. The oil/water ratio was set to 5:1, avoiding the hydrogel agglomerate, and all precursor solution could be converted to Lac/particles in the meantime. Then, the amount of PEGDA/PEGMA mixture was adjusted to 67% (33% of Tris-HCl solution with Lac) based on the same considerations mentioned above. For UV polymerization, the exposure time of light and the concentration of initiator will influence the degree of polymerization. A large fraction of initiator and longer UV treating time was undesirable because these characteristics will adversely impact the enzymatic activity. Considering complete polymerization and least loss of enzyme activity, UV light exposure time was set to 3 min and the fraction of light initiator was set to 1% (1 μL light initiator in 100 μL water phase)—based on a functional biomaterial perspective. 

In this study, 200 µL of the precursor solution was added to 1 mL of light mineral oil with 1% Span 80 and 1% Tween 20 and vortexed for 10 s to achieve a well homogenized emulsion. Then, the emulsion was polymerized by exposure to UV light (B-100AP, Analytik Jena AG, Jena, Germany) for 3 min and separated from the oil by repeated washing using 0.1 wt% Pluronic F-127 and citric phosphate buffer (pH 7). Subsequently, GA was added to the Lac/particles with the final concentration of 0.5% (v/v), and chemical cross-linking was performed at room temperature under shaking for 30 min. The excessive aldehyde groups were capped by incubation in the Tris-HCl buffer for 30 min. The produced Lac/particles were stored at 4 °C until used. 

The surface morphologies and size distribution of Lac/particles were analyzed using scanning force microscopy (XL30, FEI, Hillsbor, OR, USA) and laser particle size distribution analyzer (Bettersize 2000, Baite, Dandong, China), respectively. The fluorescence of the Lac/particles was observed using a fluorescence microscope (EVOS, ABI, Foster, CA, USA). The absorbance for activity assay was determined using a UV-vis spectrophotometer (UV-2450, Shimadzu, Tokyo, Japan).

### 2.3. Labeling of FITC to Lac

The enzymes to be trapped in the hydrogel were initially fluorescently labeled to monitor the formation of the Lac/particles. Free amino groups of Lac were linked with isothiocyanate reactive groups of FITC, forming a stable thiourea bond. Then, 5 mg/mL Lac was reacted with 50 mg/mL FITC in a 0.1 M carbonate-bicarbonate buffer (pH 9.0) for 2 h in a dark room at room temperature. Then, the solution was isolated by a gel filtration column to separate Lac, which has been labeled. The conjugation of FITC to the Lac was confirmed by an UV-vis spectrophotometer (UV-2450, Shimadzu, Tokyo, Japan).

The amount of the entrapped Lac was estimated by measuring the concentration of enzymes in the initial solution or supernatant by Bradford assay. The enzyme loading yield was calculated as follows:

(1) $$\mathrm{Loading}\;\mathrm{amount}=\frac{m_{0}-m_{t}}{m_{0}}\times 100\%$$

where *m*_0_ and *m_t_* represent the initial and residual amounts of Lac, respectively. 

### 2.4. Activity Assay 

Lac activity was determined by monitoring the oxidation rate of ABTS to its cation radical (ABTS^+^) at 420 nm (ε_420_ = 36,000 L/M cm^−1^) in 50 mM citric phosphate buffer (pH 3.5) with shaking under 150 r/min at 25 °C. One unit (U) of Lac activity was defined as the amount of enzyme forming 1 μmol of ABTS^+^ per minute. All spectrophotometer measurements were performed using a Shimadzu UV-2450 (Tokyo, Japan).

### 2.5. Determination of Enzyme Kinetics 

The kinetic constants *K_m_* and *V_max_* of the free Lac and Lac/particles were determined by measuring the initial rates of the reaction with ABTS (100–500 μM) in citric phosphate buffer (50 mM, pH 3.5) at 25 °C. The *K_m_* and *V_max_* of the free and Lac/particles were calculated from the Lineweaver-Burk plot as follows:

(2) $$\frac{1}{v}=\frac{K_{m}}{V_{\max}}\frac{1}{\left[S\right]}+\frac{1}{V_{\max}}$$

where *K_m_* and *V_max_* are the kinetic constants, and [S] is the concentration of the substrate.

### 2.6. Stability of Free and Lac/Particles

The pH stabilities of free Lac and Lac/particles were tested by measuring the activity after incubation with different citric phosphate buffer solutions (pH 3–7) under continuous shaking (150 r/min). Enzymes were statically stored at 4 °C, and the residual activity of both free Lac and Lac/particles were assayed to determine the storage stability. The residual activity was expressed as a percentage relative to the initial enzymatic activity. 

### 2.7. Biotransformation of BPA 

The biotransformation of BPA was performed by addition of Lac/particles (30 mg) into 0.5 mL of BPA solution (25 mg/L) and incubating at room temperature with shaking at 150 r/min to enable a homogeneous and sufficient enzymatic reaction. 

Solvent extraction was conducted to extract residual BPA retained in the Lac/particles at the end of the incubation period. Lac/particles thoroughly mixed with 0.5 mL of methanol was sonicated for 20 min, and the supernatant was collected. This process was performed twice to extract completely. The extracted BPA in the solution was measured by HPLC analysis.

For BPA quantification, the HPLC system was equipped with a UV-vis detector at 278 nm and an Eclipse Plus C18 column (250 mm × 4.6 mm, particle size of 5 μm, Agilent, Palo Alto, CA, USA). The mobile phase was composed of acetonitrile and deionized water with a volume ratio of 50:50. The flow rate was set to 1.0 mL/ min at 30 °C. The BPA transformation efficiency was assessed as follows:

(3) $$\mathrm{BPA}A\;\mathrm{transformation}=\frac{C_{0}-C_{t}}{C0}\times 100\%$$

where *C*_0_ and *C_t_* represents the initial and residual concentrations of BPA in the reaction, respectively.

### 2.8. Reusability of Lac/particles

The reusability of Lac/particles was studied using Lac/particles and deactived Lac/particles (deLac/particles). BPA removal was performed at 14 consecutive experiments with shaking at 150 r/min. In each cycle, 30 mg of Lac/particles or deLac/particles were reacted with 0.5 mL of 25 mg/L of BPA (pH 5) at room temperature. The supernatant was decanted after 1 h incubation, and then the fresh BPA solution was added for the next round reaction. 

## 3. Results and Discussion

### 3.1. Properties of Lac/particles 

The Lac/particles were prepared by emulsion of the PEGDA/PEGMA mixture containing Lac (precursor solution, as water phase) in mineral oil phase followed by UV induced polymerization (Figure 1). Under optimized conditions, 100% of the precursor solution could be converted to Lac/particles. The recovery of enzymatic activity in the produced Lac/particles was 18.9%. One intrinsic drawback of the typical entrapment method is the physical inaccessibility of some entrapped enzymes to the substrate molecules, although they still maintained the native structures [33]. This characteristic resulted in relatively lower activity recovery. However, the recovery percentage was still higher than others reported e.g., 0.1–12.9% [41] and 7.4% [42]. 

**Figure 2:**
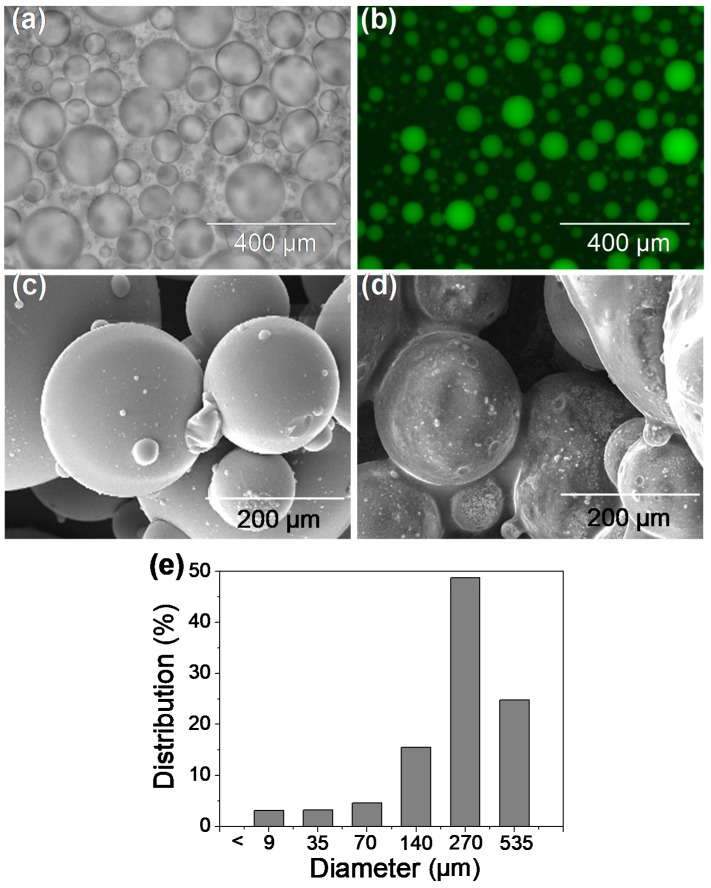
(**a**) Bright field and (**b**) fluorescent microscope images of the hydrogel droplets with fluorescein isothiocyanate (FITC) dye labeled Lac molecules entrapped inside. SEM images of hydrogel microparticles produced (**c**) without Lac entrapping and (**d**) with 100 mg/mL Lac entrapped. (**e**) Size distribution profile of the Lac/particles.

To visualize the distribution of Lac inside the hydrogel droplets, Lac was labeled with FITC fluorescent dye in advance, and the fluorescence was imaged using a fluorescence microscope. As shown in Figure 2a,b, all droplets were filled with green, which indicated the complete mixture of Lac with PEGDA/PEGMA solution, and droplet was formed without any Lac loss. No Lac-FITC was detected in the oil phase by fluorescence microscopic observation or Bradford assay, which suggested that all Lac have been entrapped in the hydrogel. The Lac/particles were nearly spherical, as shown by Figure 2c,d. The surface of Lac/particles was smooth when no Lac was entrapped in the hydrogel, but it became rough when the Lac was entrapped. This result indicated that Lac was entrapped throughout the hydrogel particles. 

The emulsion method produced micron spherical particles (2.33–535 μm diameter), and the particles size was mostly in the range of 137–535 μm, accounting for 73.54% of the total (Figure 2e) particles, with broader size distribution range. Behrens et al. synthesized N-(3-aminopropyl) meth-acrylamide (APM) hydrogel particles via inverse suspension polymerization and investigated their use as a hemostatic material. The particle size of APM hydrogel was 600–900 μm, which was bigger than ours. Most important of all, much more time (24 h) and continuous stirring were required [43], which is not suitable for enzyme immobilization. Zheng et al. prepared granular hydrogel using Fenton reagent as redox initiator under an ambient temperature in air atmosphere with acrylic acid (AA) and allylthiourea (AT) as the monomers within 2 h, but the obtained hydrogel particle size was much bigger and was thus required to be sieved by 40–80 mesh before used for removing pollutants [44]. While the emulsion electrospray technique was used to prepare PLGA microspheres, which combined with thermosensitive hydrogel, hydrogels with smaller sizes (about 5 μm) were produced, which may be appropriate for drug release but are not suitable for water treatment because smaller particle size was much more difficult for recovery [45]. Thus, the method we used is time-saving, less impact on enzyme activity, and appropriate particle size could be achieved, which was not only good for mass transfer, but also easy for reuse. The shortcoming of this work was its broader size distribution compared with microfluidic technique, in which, however, a much more complex device was required [46]. Xia et al. [47] found that the particle size distribution caused by the emulsion was much broader than by the microfluid device. However, the quantity of particles can be obtained at one time by the emulsion method, facilitating mass production. Moreover, the produced micron size particles not only resulted in the automatic sedimentation without additional centrifugation or separation but also had little effect on the substrate transport to the Lac entrapped inside the particles.

### 3.2. Kinetics and Stability of Lac/Particles

Kinetic parameters *K_m_* and *V_max_* were obtained from the Lineweaver-Burk plot using ABTS as substrate. The *K_m_* values of Lac/particles and free Lac were 1649.35 and 20.32 µM, respectively. The increase in *K_m_* for Lac/particles was due to the reduced affinity to substrate after enzyme immobilization, which was caused by the diffusion limitation given the loss of enzyme flexibility for substrate binding. The *V_max_* of Lac/particles (0.8342 μM/min) was increased by approximately 18.5 fold compared to free Lac (0.045 μM/min). In general, most enzyme carrier materials increase *K_m_*, accompanied by a decrease in the maximum rate of reaction. However, in this study, the *K_m_* and *V_max_* were increased simultaneously. The possible reason is that the adsorption capacity of Lac/particles guaranteed the substrate agglomeration on the surface of the Lac/particles and entrance to the internal to contact with the enzyme. The result was consistent with that obtained by Sun et al. [33] who entrapped the Lac into PAM-chitosan hydrogel by mechanical agitation, and *V_max_* was improved, accompanied by increasing *K_m_*.

Any changes in the chemical and physical properties of wastewater can lead to the inactivation of Lac. Thus, the stability is a crucial factor when the Lac/particles were intended to treat polluted water. In this sense, the stability profiles of Lac/particles at various pH conditions were investigated. 

**Figure 3:**
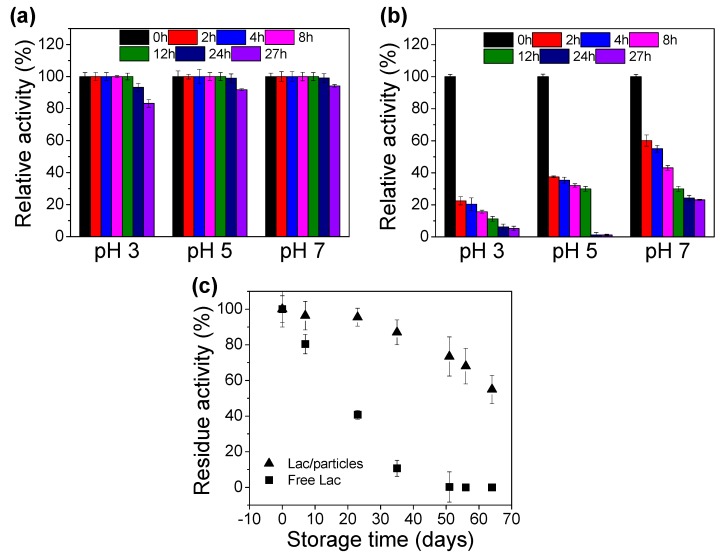
Stability profiles of (**a**) Lac/particle and (**b**) free Lac in 50 mM citric phosphate buffer solution with various pHs under continuous shaking (150 r/min) at 25 °C. (**c**) Storage stability of Lac/particles and free Lac at 4 °C.

The stability profiles of entrapped enzymes in the citric phosphate buffer at various pH levels (3, 5, 7) at 25 °C with shaking at 150 r/min were evaluated by measuring the residual activities at specific time intervals. After 2 h shaking, the remaining enzymatic activities of Lac/particles were still 100% at all pH levels (Figure 3a), and even after 12 h, the activities remained 100%. After 24 h, the activities loss was only 7%, suggesting its superior stability. In addition, the relative activities of Lac/particles were much stable at pH 5 and pH 7 than at pH 3. Instead, free Lac at pH 3, 5, and 7 were sharply decreased to 22.5%, 37.5%, and 60.1% after 2 h incubation (Figure 3b), respectively, and maintained only 6.25%, 1.25%, and 24.2% after 24 h. The relative activities of free Lac were remarkably stable in the pH 5 and pH 7, which was consistent to the Lac/particles. The Lac/particles could be stored at 4 °C for at least 23 days with little activity loss (4.53%), and free Lac lose 60% of initial activity at the same time, as indicated in Figure 3c. The activities of Lac/particles gradually decreased to 54.91% after 64 days. The result was markedly better than immobilized Lac on polyacrylonitrile-biochar composite nanofibrous membrane or PAN nanofibrous membrane, where the residual activities were 78% after 20 days [48] and 92% after 18 days [49], respectively. Additionally, the storage stability was superior to the polyacrylamide/carragennan hydrogels [50]. After 27 days of storage at 4 °C, Lac immobilized on polyacrylamide/carragennan hydrogels retained 56–80% of their initial activities, whereas the Lac/particles in this work retained round 90%. This phenomenon indicated that the entrapment of Lac in hydrogel particles enhanced the biocatalyst stability due to the fact that enzyme immobilization restricts conformation changes, which in turn prevent denaturation and increase stability. In addition, several reports have confirmed that Lac activity would be protected by adding PEG [51,52]. Thus, the PEG used as an immobilization matrix in this study may be inferred to be partially attributed to the high stability of the Lac/particle. 

### 3.3. Enzymatic Biotransformation of BPA

**Figure 4:**
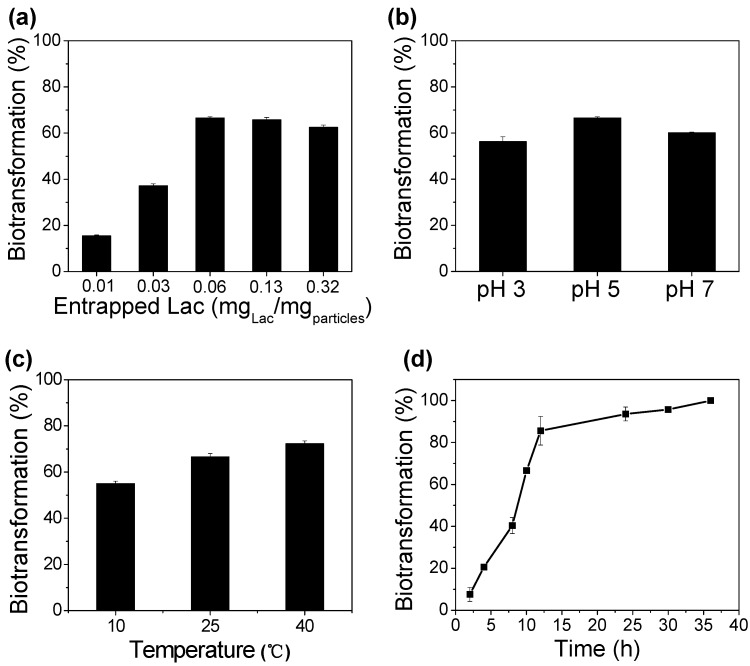
(**a**) Profiles of bisphenol A (BPA) biotransformation with Lac/particles in 50 mM citric phosphate buffer (pH 5) at 25 °C for 10 h depending on the amounts of enzymes entrapped. Profiles of BPA biotransformation with Lac/particles (0.06 mgLac/mgparticle) depending on variation of (**b**) pHs and (**c**) temperatures within 10 h reaction. (**d**) Time dependent BPA biotransformation efficiency by the Lac/particles (30 mg) at 25 °C. The BPA concentration was 25 mg/L.

The enzymatic biotransformation of BPA at various entrapped Lac concentrations was accessed. The increment of Lac loading amounts from 0.01 to 0.32 mg Lac per milligram particles accelerated BPA biotransformation efficiency by showing 15.48%, 37.19%, 66.6%, 65.76%, and 62.56% within 10 h of reaction, respectively (Figure 4a). A higher amount of enzyme will usually guarantee a faster transformation of the target substrate, but probably some of the entrapped enzymes were physically inaccessible to the substrate molecules, although they still possessed the native structures. Consequently, the highest BPA biotransformation efficiency was a compromise between enzyme amount and active sites, such that all active sites of Lac have the opportunity to contact with the substrate.

The biotransformation of BPA (25 mg/L) at various pH conditions was investigated by changing pH levels from 3 to 7 at 25 °C. The data presented in Figure 4b shows that the biotransformation efficiencies contributed by the enzyme catalyst were 56.36%, 66.59%, and 60.14% at pH 3, 5, and 7 within 10 h of reaction, respectively. The best efficiency was obtained at pH 5 in which the acidic condition ensured the enzymatic activities. This result was consistent with that obtained by Hou et al. (Hou et al., 2014b), who also achieved the best treatment of BPA at pH 5. However, Lac/particles have a broader pH range, because even in neutral conditions, 60.14% of BPA could be biotransformed, which is 6.45% lower than that at pH 5. 

The enzymatic biotransformation of BPA at different temperatures was also accessed by changing reaction temperatures from 10 °C to 40 °C. As shown in Figure 4c, the Lac/particles showed BPA biotransformation efficiencies of 55.04%, 66.59%, and 72.27% at 10 °C, 25 °C, and 40 °C, respectively, after 10 h of catalytic reaction, suggested a broad working temperature range. Additionally, it was demonstrated that Lac/particles have high enzyme stability and remediation applicability even at warmer or cooler conditions. 

Lac/particles (30 mg) were added into BPA solution (25 mg/L, 0.5 mL, pH 5.0), and the resulting solution was incubated at room temperature with shaking at 150 r/min to make a homogeneous and sufficient enzymatic reaction. The increment of incubation time accelerated BPA biotransformation efficiency during the first 12 h, indicating almost 85.6% BPA biotransformation (Figure 4d). Then, the efficiency was slowly increased to 93.6% within 24 h and 100% within 36 h. 

### 3.4. Synergistic Removal of BPA 

To understand the detailed mechanism of BPA removal process by the Lac/particles, a series of control experiments were conducted by treating BPA (25 mg/L) with the free Lac and the deLac/particles. The fates of the BPA, i.e., residual BPA in the liquid phase, adsorbed BPA in Lac/particles, and enzymatically biotransformed BPA, were accessed, respectively, after 24 h of reaction at 25 °C with shaking (150 r/min) condition.

**Figure 5:**
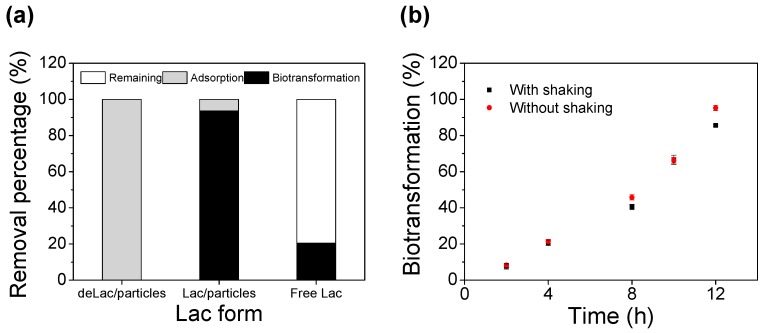
(**a**) BPA removal profiles with deLac/particles, Lac/particles and free Lac in pH 5 with shaking (150 r/min) for 24 h at 25 °C. (**b**) Comparison of BPA biotransformation efficiencies by the Lac/particles with shaking (black dot) and without shaking (red dot) at 25 °C. The BPA concentration was 25 mg/L.

As shown in Figure 5a, when BPA was reacted with the deLac/particles in which enzymes were deactivated previously, there was no remaining BPA detected in the solution, meaning BPA was all retained inside the deLac/particles due to the high efficiency adsorption. When the Lac/particles were used for BPA removal, the BPA biotransformation efficiency was 93.68% and the adsorbed portion was 6.32%, suggesting that BPA was firstly adsorbed into the Lac/particles and then the adsorbed ones were further biotransformed by the enzyme catalytic reaction. 

As free Lac was utilized for BPA removal, 20.41% of BPA was biotranformed that was much lower than with the Lac/particles. Inadequate contact between Lac and the substrate is usually one of the obstacles for efficient micropollutants biotransformation. The reason for the enhancement of BPA biotransformation efficiency by the Lac/particle in this study was firstly due to its great adsorption capability that can concentrate BPA inside the Lac/particles, allowing easy and fast mass transfer, promoting the interaction between BPA and active sites of enzymes, and facilitating efficient catalytic reaction. Another reason was that the Lac enzyme molecules entrapped in the PEG hydrogel microparticles may be less affected by the environmental changes compared to the free one, and the activity loss during reaction was avoided. 

To investigate whether the mixing process is necessary during catalytic degradation of BPA by using Lac/particles, the BPA biotranformation profiles at continuous shaking and no-shaking conditions were compared. It was found that the Lac/particles (30 mg) can adsorb all of the BPA in the solution within 30 min, and no BPA could be detected in the supernatant. This phenomenon confirmed that all BPA had been adsorbed into the Lac/particles. Thus, for the no-shaking experiment, the supernatant solution was decanted after 30 min, and the Lac/particle that was enriched with BPA was statically incubated at 25 °C for catalytic reaction. For continuous shaking experiment, BPA solution containing Lac/particles were continuously shaken (150 r/min) during the whole reaction process without decanting the supernatant. As shown in Figure 5b, the enzymatic biotransformation profiles between the two different modes were almost overlapped. This result demonstrated the continuous mixing process, which was a generally required condition in other reports, was not necessary in this study, which was attributed to the fast and efficient BPA adsorption ability and the great catalytic reaction capability of the Lac/particles. Additionally, as another point of view, this result indicated that the BPA biotransformation reaction was conducted inside the Lac/particles independent of the surrounding buffer solution so the BPA adsorbed inside. Therefore, the benefit of Lac/particles in BPA removal was prospective, as this process combined BPA adsorption and enzymatic oxidation, and the adsorption ability made the operation more efficient, easy, and economic compared to the previous report [53].

### 3.5. Reusability of Lac/Particles

**Figure 6:**
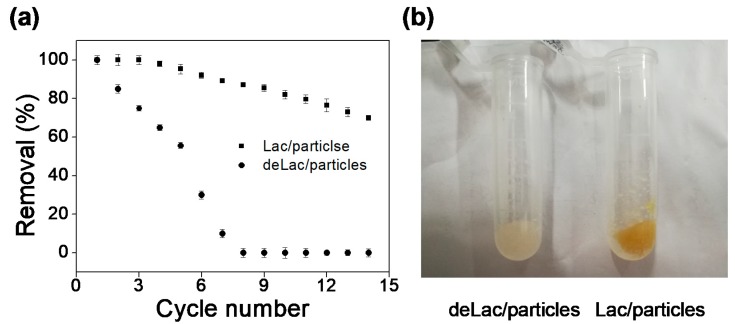
(**a**) Reusability of Lac/particles and deLac/particles as indicated by the changes in the BPA removal efficiencies. (**b**) The color change of Lac/particles and deLac/particles after repeated reaction with BPA. The BPA concentration was 25 mg/L.

The reusability of Lac should be an essential concern when it was intended to be applied for polluted water remediation. In this study, the removal efficiency of BPA by Lac/particles and deLac/particles were investigated by repeated usage for treating fresh BPA solution for 14 cycles. The BPA removal efficiencies were both 100% for the first cycle, and the enzymatic biotransformation functioned in the following cycles (Figure 6a). The difference in the removal efficiencies between the two forms of Lac was obvious during the cycle process, and this characteristic suggests a great improvement in reusability efficiency of Lac/particle. Due to the adsorption of BPA, the sites were saturated gradually for the deLac/particles. However, the adsorption sites on Lac/particles can be liberated, because Lac entrapped in the particles can catalyze the BPA, and the cycle of adsorption-biotransformation could be restarted. Therefore, the entrapped Lac enabled the regeneration of adsorption sites, preventing the total saturation of Lac/particles and allowing reuse. These features are necessary if a continuous operation mode is desired. The decrease in BPA removal by the Lac/particles in the later cycles may be due to aggregation of the product, which resulted in the Lac activity loss and occupying the active sites. The color of Lac/particles after 14 cycles became much deeper than the deLac/particles (Figure 6b) because the enzymatic catalytic product was formed, accumulated, and captured in the Lac/particles. For deLac/particle, no enzymatic oxidation product was generated, only adsorption functioned. Hence, the color of the deLac/particles were still similar to the initial.

In all experiments, the Lac/particles maintained their shape during the whole reaction. We also evaluated the release of Lac from the particles under all reaction conditions, including all pH levels, and no leakage of Lac from the particles was detected by Bradford method. 

## 4. Conclusions 

Spherical and micron sized Lac/particles were prepared via UV assisted emulsion polymerization, which was suitable for mass production of enzyme entrapped hydrogel miroparticles with high enzyme loading, advantageous mass transfer, and convenient recovery. The Lac/particle was much more stable and presented fast adsorption and efficient biotransformation profiles toward BPA. External shaking was not a necessary factor due to the conducting of the enzymatic BPA removal inside the micro reacting environment of the Lac/particles. The Lac/particle could be reused more than 10 times with little decrease of removal efficiency. The combination of adsorption and enzymatic biotransformation of the Lac/particle is promising for fast and cost-effective BPA remediation.
